# Building functional and sustainable pharmacovigilance systems: an analysis of pharmacovigilance development across high-, middle- and low-income countries

**DOI:** 10.1177/20420986251342941

**Published:** 2025-06-10

**Authors:** Olga Menang, Peter van Eeuwijk, Karen Maigetter, Andrea de Soyres-Kuemmerle, Edinam Agbenu, Christian Burri

**Affiliations:** Department of Medicine, Swiss Tropical and Public Health Institute, Kreuzstrasse 2, Allschwil 4123, Switzerland; University of Basel, Basel, Switzerland; Department of Epidemiology and Public Health, Swiss Tropical and Public Health Institute, Allschwil, Switzerland; University of Basel, Basel, Switzerland; Department of Epidemiology and Public Health, Swiss Tropical and Public Health Institute, Allschwil, Switzerland; University of Basel, Basel, Switzerland; Department of Medicine, Swiss Tropical and Public Health Institute, Allschwil, Switzerland; University of Basel, Basel, Switzerland; World Health Organisation Regional Office for Africa, Ouagadougou, Burkina Faso; Department of Medicine, Swiss Tropical and Public Health Institute, Allschwil, Switzerland; University of Basel, Basel, Switzerland

**Keywords:** challenges, functionality, healthcare system, LMIC, pharmacovigilance, strengthening, sustainability

## Abstract

**Background:**

There has been notable progress in pharmacovigilance (PV) in low- and middle-income countries (LMIC) in the last decade. However, only a few of these PV systems are fully functional, unlike in high-income countries where stringent legislation, regulations and operational guidelines have enabled the establishment of effective PV systems. The key challenges faced in LMIC include organisational inefficiencies; weak infrastructure; inconsistent and poorly enforced regulations; and inadequate financing and shortage of trained personnel. Furthermore, low adverse event volume and poor data quality hinder the capacity for safety data generation and utilisation. With the increasing availability of essential and innovative medicines in LMIC, establishing robust PV systems is crucial for effective safety surveillance.

**Objectives:**

This research aims to analyse the development of PV systems across high-, middle- and low-income countries and to carve out essential elements for functionality and sustainability of PV systems in LMIC.

**Design:**

A convergent parallel mixed-methods design, combining qualitative and quantitative methods.

**Methods:**

Qualitative and quantitative research consisted of semi-structured interviews and an online survey, respectively.

**Results:**

Twelve key informants from 10 countries were interviewed and 52 respondents from 36 countries completed the online survey. From the qualitative and quantitative data, we identified nine essential elements for sustainable PV development in LMIC: understanding the drivers of PV development; adequately resolving core system challenges; implementing an efficient organisational structure and good governance; establishing procedures for PV activities; ensuring availability of qualified and trained staff; identifying alternate sources of financing; having a strategic development plan; adequately leveraging the health system; and effectively integrating the pharmaceutical sector in the national PV system.

**Conclusion:**

Findings from this research indicate that significant efforts are still needed to upgrade PV systems in LMIC to meet global standards despite the progress achieved in recent years. Developing the different areas emerging from this research, within the framework of a holistic, fit-for-purpose PV system strengthening, would enable a comprehensive progression from basic to functional and thus sustainable PV systems in LMIC.

## Plain language summary

Understanding how systems to monitor the side effects of authorised medicines are established and maintained in different parts of the world in order to identify and propose ways to build these systems in developing countries

*Why was the study done?* Despite notable progress in the last decades in many developing countries, the systems that monitor the side effects of approved medicines are still not fully functional. Challenges include poor organisation, weak infrastructure, inconsistent regulations, lack of funding and trained staff. With increased access to medicines in developing countries, it is crucial for these countries to strengthen the monitoring of medicine side effects to prevent harm. This research aims to understand how side effects of approved medicines are monitored in different parts of the world, in order to propose ways to enhance surveillance in developing countries.

*What did the researchers do?* We combined two research tools: interviews followed by an online survey to broaden the knowledge gained. Twelve experts from ten countries were interviewed and 52 people from 36 countries completed the online survey.

*What did the researchers find?* We identified nine important elements for long-lasting and effective growth of systems to monitor side effects of medicines in developing countries. These are: understanding what motivates experts; finding durable solutions to key shortcomings of the systems; establishing efficient organisation and structures; putting in place standard steps to collect, analyse and use the information collected; ensuring that dedicated staff are knowledgeable and well-trained; identifying stable sources of financing; maintaining a development plan; making use of the health system; and involving companies that produce medicines.

*What do the findings mean?* This study shows that although systems to monitor side effects in many less developed countries have improved, further work is still needed to upgrade them to meet global standards. The nine important elements identified in this research, if implemented, can support a gradual and logical improvement of the monitoring of side effects in developing countries.

## Background

Pharmacovigilance (PV) is an essential component of healthcare systems, with the objective to prevent harm from adverse drug reactions (ADR) and promote the safe and effective use of medicinal products.^1^ Despite great advancements in PV since the thalidomide disaster in the 1960s,^2,3^ ADR remain an important cause of morbidity and mortality, with a huge economic burden on health systems globally. Several meta-analyses and studies have estimated the frequency of ADR-related hospitalisation between 2.3% and 10% of hospital admissions in developed countries.^4–7^ In the United States and Europe, the financial burden is estimated at US $30.1 billion and €79 billion, respectively.^8,9^ The global burden of ADR is often underestimated because of limited global epidemiological studies and data mainly originating from developed studies and from hospital settings.^7,8,10^ This highlights the importance of robust PV systems for early identification and evaluation of safety signals, and timely risk communication to healthcare professionals (HCP) and the public.^11–13^

In high-income countries (HIC), PV has evolved into a highly regulated discipline with legislation, regulations and guidelines to establish robust and performant PV systems.^14^ In response to a 2008 report estimating that in the European Union (EU) approximately 5% of all hospital admissions and 197,000 deaths per year were caused by ADR,^15^ the new PV legislation was adopted in 2010.^16,17^ The EU PV system is one of the most advanced globally, based on a regulatory network consisting of the National Regulatory Authorities (NRAs) of member states, the European Medicines Agency (EMA) and the European Commission.^18^ The EMA acts as a decentralised agency of the EU, responsible for the scientific evaluation, supervision and safety monitoring of medicines in the EU. Each NRA, marketing authorisation holder (MAH) and the EMA must operate a PV system and shall establish and use an adequate and effective quality system for performing their PV activities. Practical measures to facilitate the performance of PV in accordance with the legislation are provided in the Guideline on Good Pharmacovigilance Practices, a set of measures drawn up to facilitate the performance of the safety monitoring of medicines in the EU.^19^

In low- and middle-income countries (LMIC), assessments of PV systems in the past decades revealed basic and weak PV systems, inadequately equipped to address medicine safety-related issues.^20–22^ When PV was developing in HIC, growth was slow in LMIC, probably because access to essential medicines was the priority. PV was introduced through Public Health Programmes (PHP) in many LMIC and its development was intricately linked to these PHP. It is only in recent years that PV is developing as a regulatory function with countries implementing holistic PV systems guided by validated tools such as the World Health Organization (WHO)–Global Benchmarking Tool (GBT).^23^ Although numerous interventions aimed at building PV capacity in LMIC have been implemented in LMIC,^24^ the PV landscape in LMIC has evolved only in the last years. Most countries now have a legal framework and an organisational structure for PV,^25^ and others such as Eritrea, Morocco, Jordan and Ghana, demonstrate PV regulatory functions in line with current international standards.^26–30^ Nevertheless, as of December 2024, only 15 LMIC have attained WHO-GBT performance maturity level (ML) 3 on a scale of 4, that is, a well-functioning and integrated regulatory system.^31,32^ Core PV challenges reported in LMIC include over-reliance on development partners, limited financial and human resources, high PV staff turnover, poorly enforced regulations and guidelines, insufficient national coordination, low ADR volumes, poor data quality and inadequate PV tools.^25,33–36^ This results in a limited capacity to generate signals and inability to utilise local data to evaluate the benefits and risks of authorised medicines.

There are several reasons why effective PV is necessary for healthcare systems in LMIC. Firstly, PV systems and medicine use are influenced by factors that can affect the safety profile of authorised medicines such as the healthcare system, the socio-political and economic climate, as well as local ecological and genetic factors.^37,38^ Secondly, pharmaceutical development in LMIC is fast growing with newly qualified manufacturers needing PV guidance.^39,40^ Thirdly, there is increased access to essential and novel medicines in LMIC, for example, malaria vaccines. In addition, with the simultaneous launches of new medicines across the globe, for example, COVID-19 vaccines, LMIC regulators will not be able to fully rely on the safety and risk–benefit assessments as they are conducted in developed countries. The historical ‘one-size-fits-all’ approach to develop PV in LMIC, that is, to establish minimum capacity for passive surveillance in all countries and develop advanced PV methods (e.g. signal detection and active surveillance) in countries where new vaccines are introduced,^41^ laid the foundation for PV development in LMIC but did not succeed to establish functional and autonomous PV systems. Consequently, in the last decade, there has been a paradigm shift towards a more holistic system-driven capacity building, such as the Smart Safety Surveillance (3S) concept, with positive results.^24,42^

Many studies have aimed to assess PV structures, processes and outcomes using validated tools such as the WHO-GBT.^23^ Likewise, numerous strategies have been proposed to build and strengthen PV systems in LMIC.^24^ Nevertheless, many LMIC need optimal and feasible mechanisms to establish functional and sustainable PV systems. This research builds on findings of a scoping review on strategies and interventions to strengthen PV systems in LMIC.^24^ It aims to analyse stakeholders’ perspectives on developing PV systems across high-, middle- and low-income countries and to propose essential elements and mechanisms to build and maintain robust and sustainable PV systems in LMIC.

## Methods

### Study design

**Figure 1.**
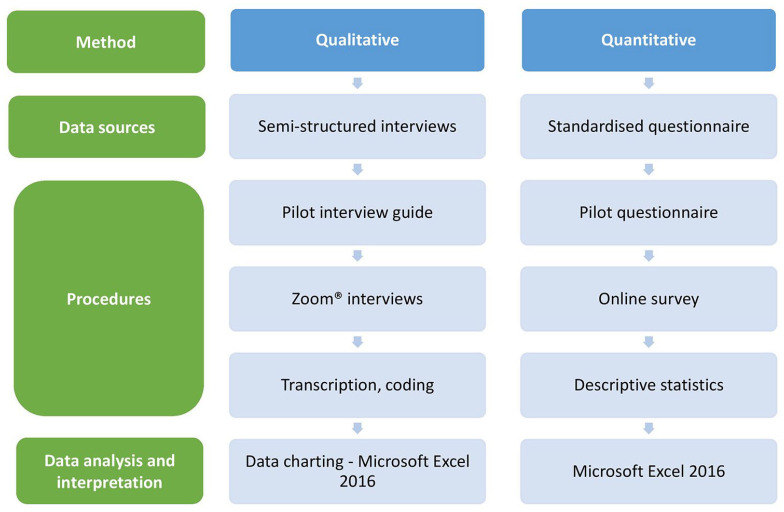
Convergent parallel mixed-methods design.

The study had a convergent parallel mixed-methods design, consisting of qualitative and quantitative methods (see Figure 1). Qualitative research contained semi-structured interviews. To expand the breadth and depth of understanding and corroboration of the study, a quantitative survey was conducted, focusing on the same thematic questions as the semi-structured interviews. Combining in-depth information from interviews with quantifiable data from the survey enabled a more complete understanding of building functional PV systems. Furthermore, by triangulating these two methods, weaknesses in either method were mitigated and complemented with the strengths of each other to enhance the validity and reliability of study results.

### Sampling, setting and study population

The study population consisted of national and global PV stakeholders, including representatives from the NRA, PHP, such as national immunisation programmes, technical and donor agencies (hereafter referred to as Technical and Financial Partners (TFPs)), from high-, middle- and low-income countries.

For the interviews, countries were selected based on the publicly available information corresponding to their PV MLs,^23^ such that all PV MLs were adequately represented. Potential participants were contacted via email addresses provided by their organisations or via regional PV mailing lists. In addition, authors of articles included in a scoping review of strategies to build PV in LMIC, conducted within the context of this research, were contacted through the email addresses provided.^24^ At least one LMIC from each WHO Region was identified.^43^ Sampling was purposive, based on informants’ knowledge and expertise in PV and their decision-making position within the national and global PV organisations concerned. For the qualitative research, it was deemed sufficient to interview 8–12 key informants, based on evidence suggesting that saturation can be achieved in a narrow range of interviews, particularly in studies with relatively homogeneous study populations and narrowly defined objectives.^44^ In this study, thematic saturation was achieved after eight interviews.

For the survey, the identification of countries and the contact and selection of participants were the same as for the interviews. The number of participants was determined based on full membership of the WHO Programme for International Drug Monitoring (PIDM) at the start of the research (i.e. approximately 90 out of 131 LMIC), indicating that at least the minimum requirements for a functional PV system were present.

For both qualitative and quantitative research, the definite sample was determined by the willingness of potential informants to participate in the research. For this study, the countries were categorised according to World Bank Group country classifications.^45^

### Data collection

Qualitative data were collected through semi-structured interviews. An interview guide (Supplemental File 1) was developed based on the study’s objective and the previously performed scoping review.^24^ The interview guide was divided into 5 sections with 30 questions: (1) key informant’s role in the national PV system; (2) organisation of the national PV system; (3) PV and health system development; (4) PV and pharmaceutical development and (5) ensuring functional and sustainable PV systems. The interview guide was reviewed by the co-authors for clarity and validity of questions, then pilot-tested by a PV expert not directly involved in the research. Sixteen key informants were invited by email to participate in the interviews and were provided with an overview of the research. Verbal informed consent to record the interview and to make use of the information provided by the key informant in the research was obtained at the start of each interview. The interviews were conducted by the first author using Zoom videoconferencing (Zoom Video Communications Inc., San Jose, CA, USA) over a 9-month period (from November 2021 to July 2022). The duration of the interviews ranged from 60 to 90 min.

Quantitative data were collected using a standardised questionnaire (Supplemental File 2), which followed the interview questions. The questionnaire was developed in MS Word and set up in ODK (https://getodk.org/). A PV expert not directly involved in the research pilot-tested the questionnaire to ensure consistency and accuracy. The questionnaire, in both English and French, was distributed to 80 persons by email and WhatsApp Messenger (WhatsApp Inc., San Diego, CA, USA) between November 2022 and February 2023; three reminders were sent to non-respondents.

### Data analysis and interpretation

The Framework Method was used for the thematic analysis of qualitative data.^46^ The respondents’ statements from the interviews were verbatim transcribed by the first author, and deductive coding was used to code the transcript. For this purpose, a codebook was developed with the predefined codes organised into corresponding categories based on the research objectives (Supplemental File 3). The codes were assigned to the transcribed data, line by line, and related sub-codes were created to improve the accuracy of the analysis; then, the codes were clustered into categories. Using Microsoft Excel (Microsoft Corporation, 2016), each transcript was summarised and the data were ‘charted’ into a matrix consisting of sub-codes, codes and categories (Supplemental File 4). The data were interpreted by identification of patterns, relationships, differences and similarities leading to new thematic groups.

Quantitative data collected in ODK were exported into Microsoft Excel for analysis using the PivotTable (Microsoft Corporation, 2016). Data analysis consisted of descriptive statistics, primarily frequencies and percentages for categorical variables.

The Checklist for Mixed Methods Research Manuscript Preparation and Review^47^ was consulted when preparing the manuscript (see Supplemental File 5 for the completed checklist).

## Results

### Characteristics of interview informants

Twelve key informants from 10 countries responded positively to the invitation and were interviewed (see Table 1(a)). Fifty-eight percent (*n* = 7) of the interviewees were from sub-Saharan Africa (SSA). Ten out of 12 (83%) had at least 11 years of PV experience, and 67% (*n* = 8) worked for the NRA and the National Pharmacovigilance Centre (NPVC; see Table 1(b)). All informants affirmed that they were in a position to influence, to some extent, decisions on the development of the corresponding national PV system.

**Table 1.** (a) Countries participating in the interviews.

WHO region	World Bank country classifications by income	Country	Number of respondents
African	Low	Burkina Faso	1
		Democratic Republic of the Congo	1
		Eritrea	1
		Malawi	2
	Lower-middle	Côte d’Ivoire	1
	Upper-middle	Ghana	1
Americas	High	Chile	1
European	High	Switzerland	2
	Upper-middle	Kazakhstan	1
South-East Asia	Lower-middle	India	1

(b) Characteristics of interview informants.*	
Characteristics	Number (%)
Organisation	
Academia	1 (8)
NIP	1 (8)
NPVC	3 (25)
NRA	5 (42)
TFP	2 (17)
Position	
Chief Regulatory Officer	1 (8)
**National PV Focal Point**	1 (8)
NIP PV Head	1 (8)
NRA Chairperson	1 (8)
Pharmacist	1 (8)
Regulatory Officer	1 (8)
Technical Advisor	1 (8)
PV Technical Officer/Senior Officer	2 (17)
Head of PV	3 (25)
Years of PV experience	
**6**–**10**	2 (17)
**11**–**15**	5 (42)
**>16**	5 (42)

* Reported position rephrased and similar terms grouped for ease of presentation.

NIP, National Immunisation Programme; NPVC, National Pharmacovigilance Centre; NRA, National Regulatory Authority; PV, pharmacovigilance; TFP, Technical and Financial Partner (agencies providing financial and technical support).

### Characteristics of survey respondents

Fifty-two respondents (65%) from 36 countries completed the questionnaire (see Table 2(a)). Thirty-eight (73%) of the informants were from SSA. There were 19, 21, 10 and 2 from low-, lower-middle-, upper-middle- and high-income countries, respectively. Participants’ PV experience ranged from less than 1 to over 16 years, and 60% (*n* = 31) worked for the NRA and the NPVC (see Table 2(b)).

**Table 2.** (a) Countries participating in the survey.

WHO region	World Bank country classifications by income	Country	Number of respondents
African	Low	Burkina Faso	3
		Burundi	1
		Central African Republic	1
		Democratic Republic of the Congo	3
		Guinea Bissau	1
		Liberia	1
		Malawi	2
		Mali	2
		Mozambique	1
		Niger	1
		Sierra Leone	2
		Togo	1
	Lower-middle	Angola	1
		Benin	1
		Cameroon	2
		Congo	1
		Côte d’Ivoire	1
		Guinea	1
		Kenya	1
		Mauritania	1
		Nigeria	2
		São Tomé and Príncipe	1
		United Republic of Tanzania	2
		Zimbabwe	1
	Upper-middle	Gabon	1
		Namibia	3
Americas	High	United States	1
	Upper-middle	Brazil	3
		Mexico	1
Eastern Mediterranean	Lower-middle	Morocco	1
European	High	Switzerland	1
	Upper-middle	Kazakhstan	1
South-East Asia	Lower-middle	India	2
		Nepal	2
Western Pacific	Lower-middle	Philippines	1
	Upper-middle	Malaysia	1

(b) Characteristics of survey respondents.*	
Characteristics	Number (%)
Organisation	
Other	1 (2)
Industry	2 (4)
Academia	5 (10)
NIP	5 (10)
TFP	8 (15)
NPVC	11 (21)
NRA	20 (38)
Position	
Communication Expert	1 (2)
Head of Department	1 (2)
National Safety Committee Chairperson	1 (2)
PV Intern	1 (2)
PV Manager	1 (2)
Senior Lecturer	1 (2)
Team Leader	1 (2)
Medical Advisor	1 (2)
Not reported	2 (4)
Pharmaceutical Advisor	2 (4)
Professor in Pharmacology	2 (4)
PV Coordinator	3 (6)
PV Technical Officer/Senior Officer	4 (8)
National PV Focal Point	4 (8)
NIP PV Focal Point	4 (8)
Regulatory Officer	5 (10)
Scientist/Senior scientist	6 (12)
Head of PV	12 (24)
Years of PV experience	
<1	3 (6)
>16	9 (17)
**11**–**15**	12 (23)
**2**–**5**	12 (23)
**6**–**10**	16 (31)

* Reported position rephrased and similar terms grouped for ease of presentation.

NIP, National Immunisation Programme; NPVC, National Pharmacovigilance Centre; NRA, National Regulatory Authority; PV, pharmacovigilance; TFP, Technical and Financial Partner (agencies providing financial and technical support).

### Themes, categories and codes emerging from the qualitative data

Twelve categories, clustered into four major themes based on the objectives of the interviews, emerged from the qualitative study (see Table 3).

**Table 3.** Themes, categories and codes emerging from the qualitative data.

Themes	Categories	Codes
Establish an effective PV organisation and good governance to ensure functionality and sustainability	Triggers and motivation for PV	Medicine-related safety issues
		Global PV trend
		WHO PIDM
		NRA assessments using the WHO-GBT in LMIC
	Core PV challenges	Initial challenges in HIC and LMIC
		Persisting core initial challenges
		Emerging challenges in both HIC and LMIC
	Organisation and structure for PV	Infrastructure and operations
		WHO-GBT maturity level
		Staff competency
	Stakeholder coordination	Mapping of stakeholders
		Aligning donors to national PV plan
	Priorities for PV capacity building	Reporting of ADR
		Number of advanced PV activities
	PV system financing	Dependency on external funds
		Hybrid financing by countries and donors
		Alternate internal sources of financing
	Strategic plan for systematic approach	Strategic plan or roadmap for PV development
		Mechanisms to evaluate and improve
Establish and implement standardised PV processes	Procedures for conducting PV activities	PV regulations and guidelines
		Standardised PV procedures
		Impact of digitalisation and vaccine introduction
Adequately leveraging the health system for PV development	Influence of the healthcare system on PV	PV at different levels of healthcare system
		Role of Public health programmes (PHP)
	Leveraging the health system for PV in LMIC	Health system building blocks and PV growth
Engaging the pharmaceutical sector	The role of the pharmaceutical sector in PV	Pharmaceutical sector and PV in LMIC
		Mandatory reporting of ADR
	Engaging MAHs in PV	Enforce legal provisions
		MAH fees for PV financing
		Foster communication and collaboration
		Pooling of resources by local companies in LMIC

ADR, adverse drug reactions; GBT, Global Benchmarking Tool; HIC, high-income country; LMIC, low- and middle-income countries; MAH, marketing authorisation holder; PHP, Public health programmes; PIDM, Programme for International Drug Monitoring; PV, pharmacovigilance; WHO, World Health Organization.

Findings of the research are presented by categories below. Additional data collected from the standardised questionnaires, where available, are integrated and presented alongside the qualitative data.

### Triggers and motivation for PV

Nine out of 12 (75%) interview informants reported medicine-related safety issues as the main triggers for establishing PV systems (e.g. ADR to antimalarials and antiretrovirals, adverse event following immunisation (AEFI) to measles and yellow fever vaccines, and falsified medicines). A global trend in PV, WHO PIDM membership and NRA assessments using the WHO-GBT were other reasons to develop PV, as one respondent reaffirmed: ‘*Inclusion of PV module in different assessments was very important in developing PV by creating visibility, stability and constant preparation for these evaluations*’ (NRA representative, HIC).

**Figure 2.**
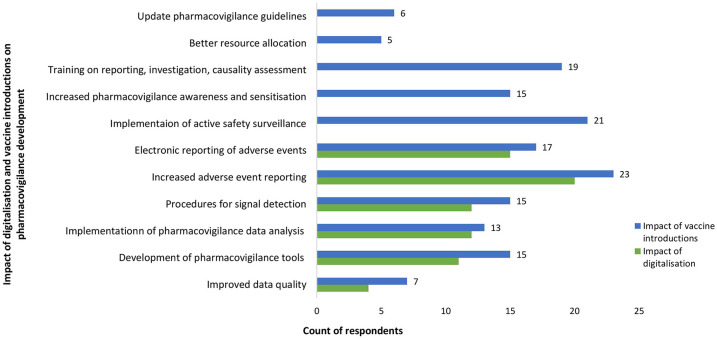
Impact of digitalisation and vaccine introductions on pharmacovigilance development in LMIC. LMIC, low- and middle-income countries.

Over 85% (*n* = 51) of study participants from LMIC reported that the two major factors that have contributed to the recent growth of PV were new vaccine introductions and digitalisation of PV processes. The impact of digitalisation and vaccine introductions on PV processes from qualitative and quantitative data is shown in Figure 2.

### Core PV challenges

**Figure 3.**
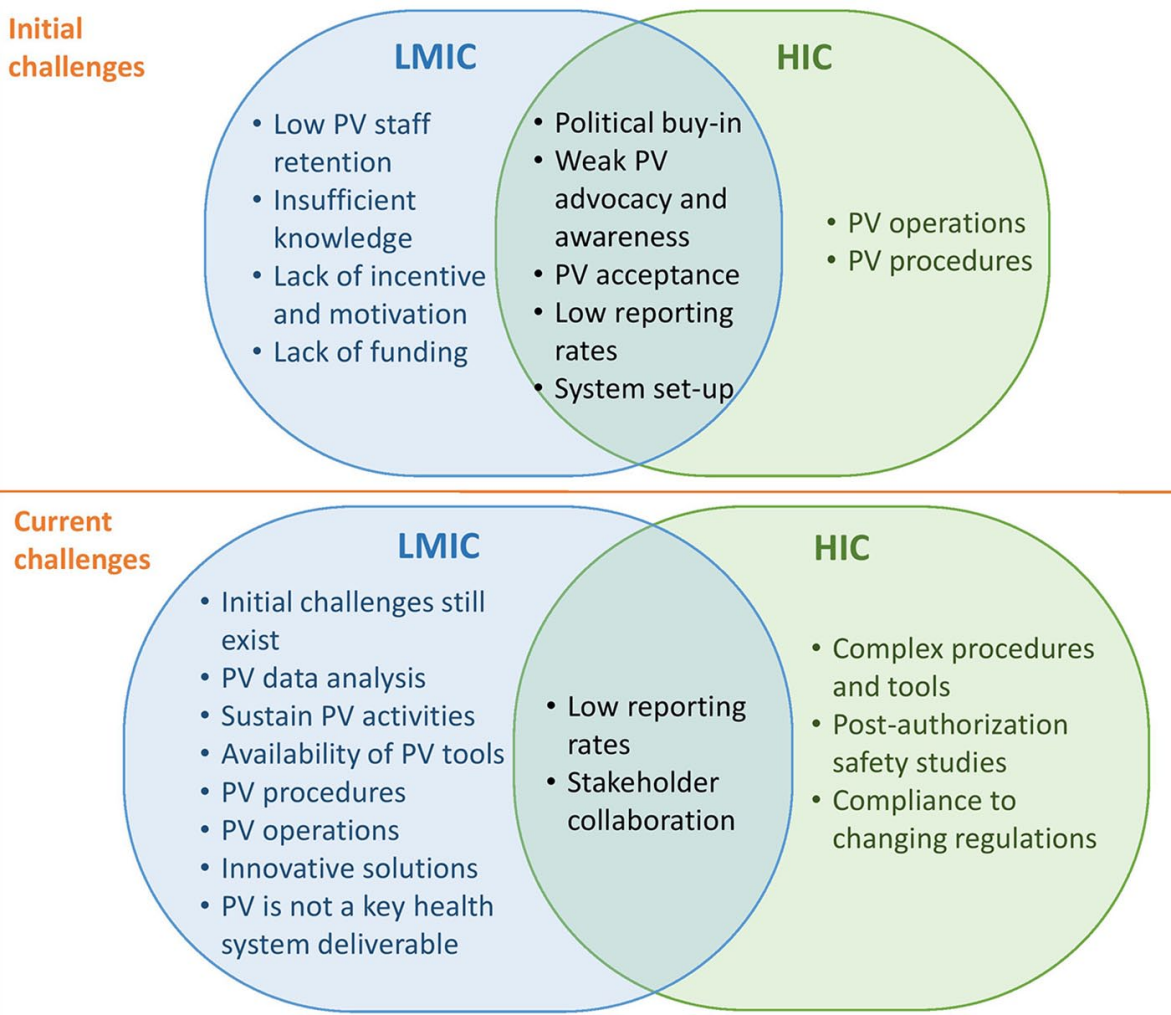
Challenges of national pharmacovigilance systems.

Across high-, middle- and low-income countries, initial PV challenges were related to obtaining political buy-in from key stakeholders, setting up different components of the PV system and creating awareness, acceptance and motivation for PV. In HIC, current challenges have evolved from initial challenges, with the major reported current challenges related to PV procedures and persistence of underreporting of ADR. On the other hand, in LMIC, core initial challenges persist. In addition, new challenges have emerged, related to effective organisation, operationalisation and routine functionality of the PV system, availability of procedures for PV activities and analysis of PV data (see Figure 3).

### Organisation and structure for PV

Interview informants reported that the national PV system either operated out of the national PV centre (e.g. India) or as a unit or department of the NRA (e.g. Chile, Côte d’Ivoire and Eritrea) or academia (e.g. The Democratic Republic of the Congo (DRC)). In all countries, there was a legal basis for PV, an organisational framework and national PV guidelines describing the roles and responsibilities of stakeholders. In some countries (e.g. DRC), regional PV centres are established to coordinate subnational PV activities. Other countries reported PV structures established at lower levels of the healthcare system, leveraging existing structures and staff, for more efficiency (e.g. Chile, Eritrea, Ghana and Malawi).

Twenty-four (46%) survey respondents from 18 (50%) countries affirmed that their countries had been either self- or formally assessed using the WHO-GBT; 15 out of these 18 countries were benchmarked at ML1 or ML2. Concerning the availability of competent and trained staff to conduct PV activities, all interview informants and 27 (52%) survey participants affirmed that staff were sufficiently competent in PV.

### Stakeholder coordination

Qualitative data indicated that PV stakeholders’ roles and responsibilities were defined in the national guidelines, and coordination was acceptable in all countries, with adequate donor alignment to the national PV strategy or policy. Likewise, 56% (*n* = 29) and 75% (*n* = 39) of the survey respondents, respectively, indicated that stakeholder coordination was acceptable and donors were aligned to the national PV plan, as indicated by an interview participant from a LMIC: ‘*Proper mapping of stakeholders is important such that efforts of donors can be harnessed not duplicated. Yes, to some extent donors influence but not significantly*’ (NRA representative, LMIC).

### Procedures for conducting PV activities

Procedures for adverse event reporting, investigation and causality assessment were developed and implemented in all countries. Every participating country reported that procedures were developed for at least one of the proposed advanced PV activities (signal detection, periodic safety update reports, risk management planning, benefit risk evaluation and active safety surveillance). All HIC, and some ML3 LMIC (Tanzania, Ghana, Eritrea, India and Nigeria), indicated that all requested procedures were implemented. The major challenge reported by interview participants was implementing and operationalising guidelines and other procedural documents as described, especially for the more advanced activities: ‘*The main challenge is implementing guidelines and operational plans, as described*’ (NRA representative, LMIC).

### Priorities for PV capacity building

Qualitative data revealed that in both HIC and LMIC, the priority for PV enhancement was training of HCP on reporting of ADR, as indicated by a representative from a NRA from a LMIC: ‘*People considered PV as a project with a start and stop date. Changing the mindset is a challenge. There is a need to constantly train and mentor*’ (NRA representative, LMIC). A participant from a HIC reported the same: ‘*AE reporting is at 1/5th of its potential. Many HCP simply do not report for lack of time, lack of interest and lack of knowledge about reporting*’ (NPVC representative, HIC).

**Figure 4.**
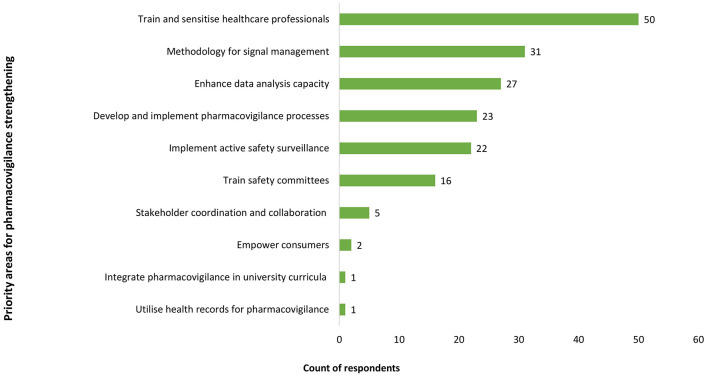
Priority areas for pharmacovigilance strengthening in LMIC. LMIC, low- and middle-income countries.

Survey findings were similar, with only 10 (19%) respondents indicating that over 50% of efforts were invested in advanced PV activities such as signal detection and periodic safety reporting. Priority areas for PV development from qualitative and quantitative data are shown in Figure 4.

### PV system financing

Interview informants from HIC reported that there were sufficient funds to finance PV activities, as did some low- (Eritrea) and upper-middle-income countries (Kazakhstan). A participant from a LMIC indicated: ‘*Yes, there is enough financing, but the issue is sustainability. How to make the system autonomous is currently under discussion as well as mechanism to generate internal funds such as registration fees and quality control fees to be used for market surveillance*’ (NRA representative, LMIC). In specific situations, Eritrea and Chile would seek external financial support, as did Ghana and India. In certain countries, a majority of PV activities were financed primarily via external support (Côte d’Ivoire, DRC and Malawi).

**Figure 5.**
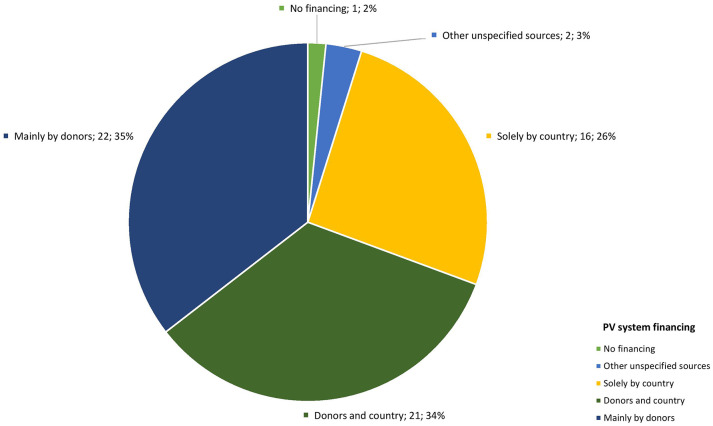
Source of financing for national pharmacovigilance systems.

Survey findings were similar, with 19 respondents (37%) indicating that PV financing was still heavily supported by external donors (see Figure 5). In fact, 34 (65%) responded that without external funding, there would be no PV activities.

### Strategic plan for a systematic approach

**Figure 6.**
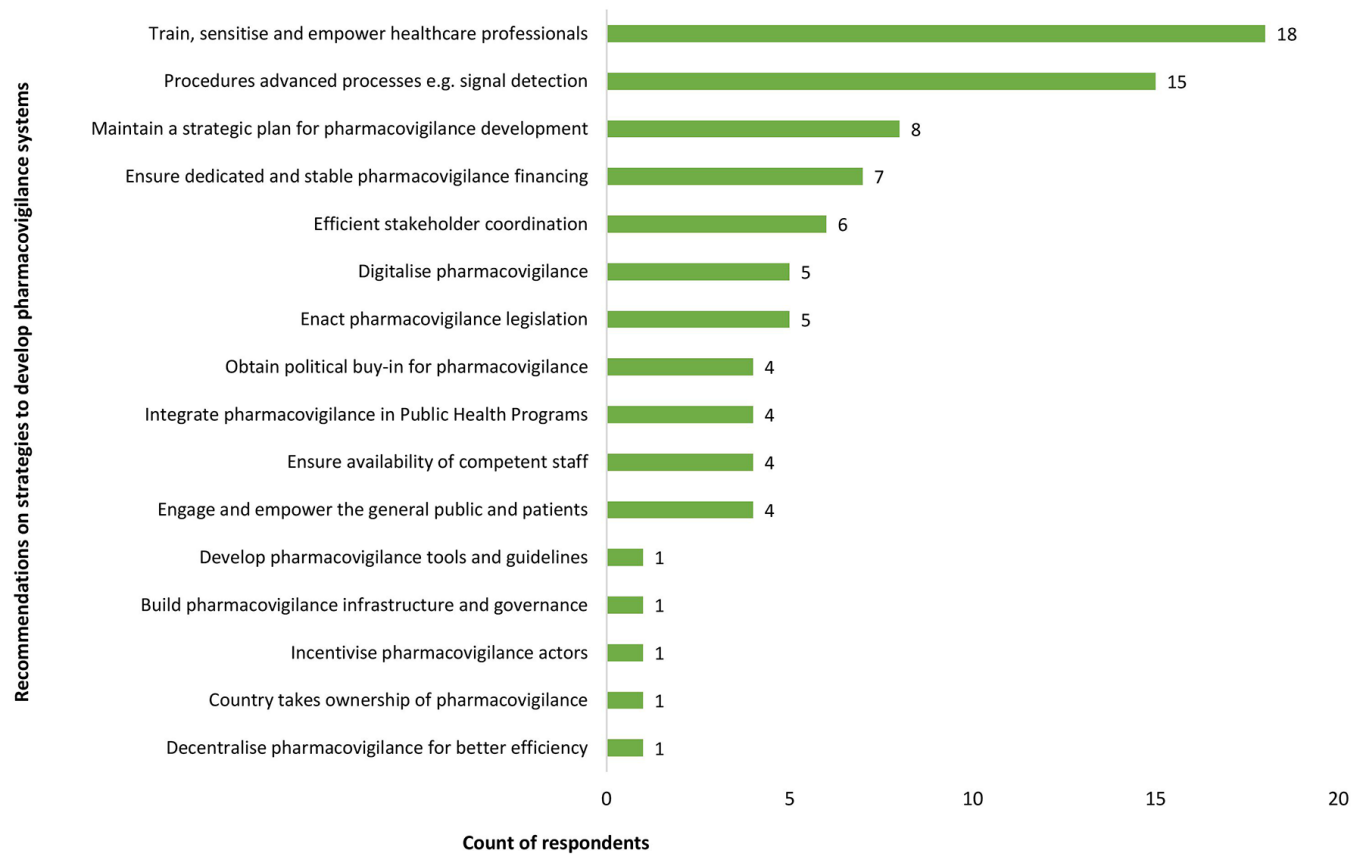
Participants’ recommendations on strategies to improve national pharmacovigilance systems in LMIC. LMIC, low- and middle-income countries.

All countries involved in the interviews and 32 survey respondents (62%) affirmed that there was a strategic plan or roadmap for PV development, often aligned with priority areas, as stated by a representative of a technical agency: ‘*Many countries have the vision and strategy but depend largely on funds, and activities are implemented as competing priorities permits*’ (TFP representative, LMIC). Overall, participants affirmed that the PV strategy was adequate, reflecting the countries’ needs, even though periodic reviews of the strategy would be beneficial. A participant from a LMIC reported that in addition to strategic plans, the NPVC had yearly operational plans as well as a weekly plan: ‘*Yes, there is a 5-year strategic plan in line with the health system. PV has been defined as a priority area by the Ministry of Health. PV is included in each PHP*’ (NPVC representative, LMIC). Participants’ recommendations on strategies to improve national PV systems in LMIC are presented in Figure 6.

### Influence of healthcare systems on PV growth

With the exception of Chile and Switzerland (HIC), all interview informants and 83% of survey respondents affirmed that the healthcare system influenced the growth of PV, as reaffirmed by numerous participants: ‘*Yes, absolutely. PV benefits from health system e.g., using differential diagnosis, background rates*’ (NIP representative, LMIC); ‘*Yes, healthcare systems support PV system; that is the way the system is structured*’ (NRA representative, LMIC). Respondents indicated that the introduction of medicines and vaccines through the PHP has been a strong enabler for PV growth in LMIC, as already presented in Figure 2.

### Leveraging the health system for PV

**Figure 7.**
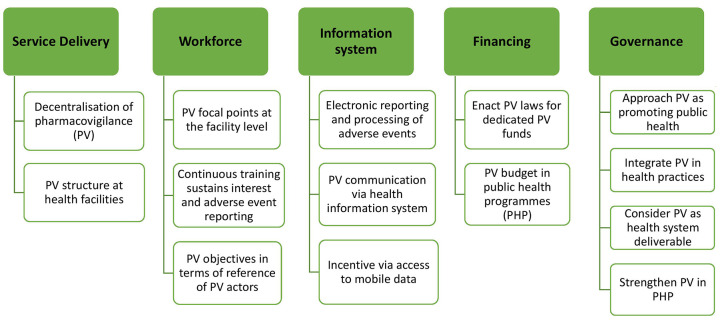
How the healthcare system acts for pharmacovigilance in LMIC. LMIC, low- and middle-income countries.

Interview informants from 8 out of 10 countries (80%) and survey respondents from 34 out of 36 countries (94%) reported a three-level healthcare system. In 18 countries (50%), PV was considered well integrated at all three levels of the healthcare system. Sixty-seven percent (*n* = 8) of interview informants and 81% (*n* = 42) of survey respondents believed that effectively integrating and promoting PV at each level of the healthcare system has enabled the development of PV: ‘*Main secret of success is the integration of PV in each facility enabling reporting to the higher levels*’ (NRA representative, LMIC). Adequately leveraging the health system structure will consequently contribute to a sustained growth of PV, as demonstrated in Figure 7.

### The role of the pharmaceutical sector in PV development

Findings from the qualitative study showed that in the opinion of a majority of interview informants, there was no correlation between pharmaceutical development and PV growth. A participant from a HIC reported: ‘*Mandatory reporting since 2002 enabled collaboration and exchange with industry and has been the main development vis-à-vis PV*’ (NPVC representative, HIC).

On the contrary, 85% (*n* = 44) of the survey respondents believed that pharmaceutical development influenced the growth of PV.

### Engaging marketing authorisation holders in PV

All interview informants and 19 (37%) survey respondents affirmed that legal provisions mandating PV and ADR reporting by MAH were implemented in participating countries. For 38 (73%) survey respondents, implementation of regulations was ongoing, although the process was challenging, as confirmed by a representative of a technical agency: ‘*Many laws and regulations have been copied from more developed countries, but how to enforce these is another question*’ (TFP representative, LMIC). The roles and responsibilities of MAH were described in national guidelines; all countries had at a minimum ADR and AEFI surveillance guidelines. The more developed LMIC, like Ghana, reported other guidelines, including PV inspection, ADR reporting and risk management planning guidelines, while HIC had guidelines for all critical PV activities. With the exception of Switzerland and Kazakhstan, interview informants reported that MAH’s collaboration with NRA was weak.

**Figure 8.**
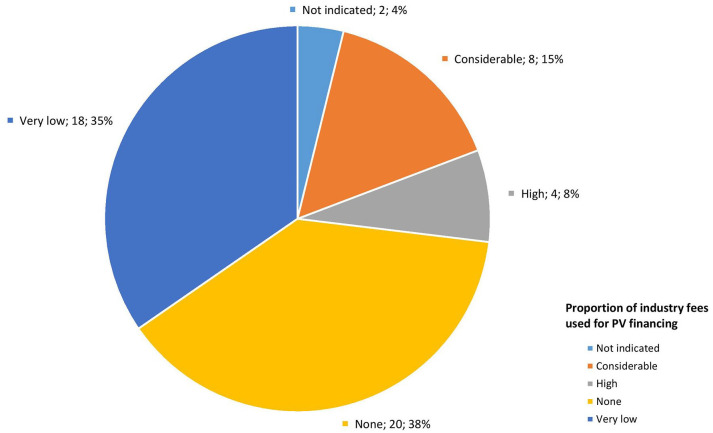
Proportion of industry fees used for pharmacovigilance financing.

Concerning PV fees, only 12 (23%) survey respondents indicated that some proportion of the fees levied on industry was used for PV (see Figure 8). All interview informants and 69% (*n* = 36) of survey respondents agreed that directing some of the industry fees to support PV activities would help to fund and establish PV: ‘*Industry pays retention fees for prospective year. It is worth considering splitting this between quality and PV*’ (NRA representative, LMIC).

## Discussion

This research aimed at describing the establishment of PV systems in high-, middle- and low-income countries, and proposing essential elements for implementing robust PV systems in LMIC. Nine essential elements were identified, pertaining to different aspects of key policy recommendations or strategies to develop PV in LMIC. They are presented below.

### Understand triggers and motivation for PV

A first step towards effective and sustainable PV is understanding the triggers for establishing a national PV system because this can provide insight into the necessary effort to maintain the system functional. For all participating countries, medicine-related safety disasters were the main triggers for establishing PV. Global awareness in PV, boosted by the COVID-19 pandemic, has reinforced the recognition that functional PV systems are also indispensable in LMIC. The WHO-GBT has been described as a game changer for strengthening national regulatory capacity^[Bibr bibr49-20420986251342941] 48^ and many LMIC are striving to attain WHO-GBT ML3, that is, stable, well-functioning and integrated regulatory systems. Findings from the current study support this affirmation, with several informants indicating that the WHO-GBT, with the resulting roadmap for system improvement, triggered and sustained interest and motivation to strengthen national PV systems. However, many LMIC still require technical and financial support from external agencies to conduct assessments, define strengthening strategies and implement interventions, reflecting a certain lack of ownership of PV by national actors.^49^ After decades of PV strengthening, LMIC should now have developed the capability to assume ownership of the organisation and functionality of their national PV system, as demonstrated by several countries, such as Ghana and Eritrea, involved in the current research. Taking ownership of the PV system and locally generated safety data is necessary to drive and sustain PV development in LMIC.

### Address core PV challenges

Likewise, understanding core challenges for PV growth is essential for developing customised plans to address the gaps. Present findings suggest that a common persisting challenge across high-, middle- and low-resource economies is the underreporting of ADR^7,50–54^ which can be addressed by continuously engaging and training HCP and the public, and by simplifying the ADR reporting process. While PV challenges in HIC have evolved over time and today are related to the evolving regulatory and technology landscape,^55–58^ PV challenges in the LMIC remain similar to the stage when these systems were established; inadequate and poorly enforced PV regulations and guidance, financial and human resource constraints, limited standardised PV processes and innovative solutions including IT, and insufficient knowledge and expertise.^25,36,59,60^ These key PV challenges are the focus of current national and international policy recommendations for PV in LMIC, which include enforcing strong PV legislation, promoting effective organisation, including ensuring adequate financing and staff, promoting national and regional collaboration, increasing awareness and improving technical knowledge.^24,61^ These challenges have persisted over time because initial PV strenthening initiatives were linked to the introduction of new products, laying emphasis on PV sensitisation and ADR reporting, with insufficient efforts invested in the other components of a functional PV system. The identification of adequate, long-term and sustainable solutions to these challenges is imperatively one of the major steps to support sustainable PV development in LMIC. This will rightly position countries to handle emerging difficulties. A holistic strengthening approach will support the identification of fit-for-purpose solutions to core system challenges as discussed below.

### Establish an effective organisation and good PV governance

A comprehensive PV system identifies and relates people (e.g. HCP, PV staff, patients), structures (e.g. NRA, PHP, NPVC, industry, advisory committees, technical agencies) and processes necessary to achieve the outcomes of PV.^62,63^ Findings of our study revealed that all countries had a legal basis for PV, an organisational framework and national PV guidelines, in line with previous research. Nevertheless, only 15 LMIC, operate at ML3,^32^ including 5 countries participating in this research – Tanzania, Ghana, Eritrea, India and Nigeria.

The successful implementation of a functional PV system relies on its organisation and governance. This kind of organisation and operation is seen in effective PV systems such as the EU PV systems.^64^ This implies defining mechanisms to enforce PV legislations and ensure compliance with regulations, including penalties for non-compliance. It also requires clearly defining roles, responsibilities and accountability, ensuring financing and trained human resources, providing adequate PV infrastructure, including information technology and data management systems, implementing procedures to execute PV activities, defining collaboration and information sharing, and implementing mechanisms to monitor and improve performance. Most countries have national or regional PV guidelines (e.g. Guideline on GPV for Arab countries), which are important tools for PV development, organisation and governance. Nevertheless, for many LMIC, operationalising national PV guidelines is a challenge.^25,26,64^ With the support of tools like the WHO-GBT or following the approach of a robust PV model such as the EU PV model, countries can gradually implement PV quality management systems in line with global standards. Saudi Arabia is an example of an HIC where PV was implemented only in 2009, based on the EU PV system; within 5 years, the PV system was fully functional and effective. Factors such as government support, adequate and sustained financing, as well as qualified and trained staff, accounted for this successful PV implementation.^64,65^ Some LMIC have also shared the experience and lessons learnt from implementing a robust PV system. For instance, in Eritrea, a low-income country, with adequate legal backing, dedicated and trained human and sufficient financial resources, a fully functional PV system was established within 9 years.^27^ Likewise, Ghana has published the development of PV from vertical PHP-driven PV to a nationwide robust system.^66^ These can act as models for other LMIC.

### Identify alternate sources of PV financing

Effective organisation and good governance necessitate the availability of stable and sufficient financial resources. In developed countries, PV is sustained when there is a dedicated budget for PV, backed by legislation. In the EU, around 86% of the Agency’s budget is derived from fees and charges, 13% from the EU contribution for public health issues and less than 1% from other sources.^67^ Individual countries also have different funding mechanisms, which can serve as a basis to identify funding models in LMIC.^68^ For instance, the Netherlands Pharmacovigilance Centre, Lareb, is an independent foundation funded by the Medicines Evaluation Board (MEB), which is the drug regulatory authority and the Ministry of Health, Welfare and Sport (VWS). The MEB’s basic sources of income are the fees for medicine for human use, defined by the VWS.^69,70^ Only a few LMIC NRA can fully fund their regulatory activities, and financing of PV activities remains a major challenge in LMIC.^71^ Findings of this research are in line with existing literature and indicate that in the majority of LMIC, there is little or no PV without external financial support and where there is a budget, allocated amounts are often insufficient.^26,33,34,72^ This model is neither stable nor sustainable and is one of the main reasons for the slow growth of PV, because when funds run out, PV activities stagnate and staff are reassigned to other projects. Identifying alternative and continued financing is crucial and indispensable for building and sustaining NRA and PV systems in LMIC. In Uganda, changes in the mechanisms of financing the National Drug Authority (NDA) (i.e. increasing the proportion of funds from government and industry) led to a decrease in reliance on external donor funding. Uganda’s NDA is now 98.25% funded through industry fees for service, with minimal contribution from donors.^73^

This research revealed that several countries, such as Côte d’Ivoire, Eritrea, Ghana and Malawi, are in the process of exploring alternative funding, for example, MAH registration fees, quality control fees or retention fees for PV. These funding mechanisms are in line with those proposed in literature, such as from commercialised services (e.g. user fees, registration and licensing) and PV tax on pharmaceutical sales.^68^ Other LMIC should take this bold step, although the mechanisms are not so straightforward. For instance, in pricing models which are based on a ‘no-loss strategy’, it will be difficult to integrate the logical ‘consumer fee’ approach (i.e. financing through fees to MAH). Also, in many LMIC, a majority of essential medicines are provided by donors such as The Global Fund and GAVI, the Vaccine Alliance, thereby limitings the possibility of levying taxes. Therefore, identifying and allocating these PV funds requires careful consideration with relevant stakeholders, appropriate political buy-in, transparency and accountability in financial operations and sustainability of funding.^68^

### Ensure the availability of qualified PV personnel

According to the EMA, achieving the required quality for the conduct of PV processes and their outcomes by an organisation is intrinsically linked with the availability of a sufficient number of competent and appropriately qualified and trained personnel.^62^ In the EU, optimal function and continuous EU PV system improvement are ensured through continuous monitoring and capacity building, including trainings, knowledge sharing and strengthening initiatives such as the Strengthening Collaboration for Operating Pharmacovigilance in Europe (SCOPE) Joint Action.^74^ Stable and adequate funding ensures the continuous availability of qualified personnel. For structures and processes to be developed and implemented, staff must be knowledgeable about PV requirements and standards, must be adequately and continuously trained and must have the appropriate facilities, including digital information technology, to carry out their tasks.^62^ In line with current knowledge, findings from this research indicated that participating countries had competent staff, though not in sufficient numbers and not sufficiently experienced in some important aspects of PV. This highlights the need for appropriate initial and continuous training of PV staff based on staff’s job descriptions, with maintenance of training plans and records and individual development plans to sustain motivation and engagement. One of the consequences of inadequate funding is the suboptimal coordination of PV activities and a high turnover of personnel assigned to PV activities.^36^ To reduce turnover and ensure the availability of dedicated PV personnel, PV staff should ideally be employed by NRA or NPVC, as is the case in HIC, rather than employed on a project basis. Continuous training and capacity building can be achieved through national PV training programmes offered by technical agencies, regulatory strengthening partnerships such as that established by WHO and Swissmedic, twinning opportunities with NRA from the global North, as well as freely available online resources.^42,61,75,76^In the last decades, numerous regional training initiatives have seen the day, focusing on building capacity in activities beyond the collection of PV data, such as signal detection and data analysis. Examples include the African Union Smart Safety Surveillance (AU-3S) programme^77^ and the South-East Asia Regulatory Network (SEARN) Vigilance Working Group.^78^

### Effectively leverage the health system for PV

In LMIC, the health systems and their PHP are key PV stakeholders. Findings of this research show that, unlike in HIC, where PV is a regulatory function, in LMIC, although most participants reported that PV operated out of the NRA or NPVC, PV was in fact built on the organisational structure of health systems and approached as a component of PHP. The healthcare systems’ building blocks, such as the service delivery, workforce, information system and financing, were shown to be essential for PV development. For instance, the availability of healthcare facilities and their workforce, particularly institutional PV focal points, is essential for integrating and promoting PV development at the lowest levels of a healthcare system. Decentralisation of the national PV system, by deploying a PV focal point or establishing a PV structure to coordinate subnational PV activities, has been reported as one of the successful experiences of PV.^25,27^ More efficient communication, accessibility to reporting tools, training and sensitising HCP and more simplified procedures were reported by Eritrea and Ghana as some benefits of decentralising PV. In summary, in a model where PV is provided to be integrated into the health system, further strengthening of health system governance and financing and effective integration of PV into the healthcare system organisation will contribute to a sustained PV development.

### Effectively engaging the pharmaceutical industry in PV

Another key stakeholder, albeit less active in PV in LMIC is the MAH. This research revealed that although many countries have legal provisions and guidelines for MAH, these were not enforced. Qualitative research participants thought that pharmaceutical development did not influence the development of PV, contrary to a majority of survey participants (85%), who believed that pharmaceutical development does influence the growth of PV, and active collaboration with MAH could promote PV expansion. This divergence in opinion is interesting and highlights the poor understanding of the important responsibility of MAH to monitor the safety of authorised medicinal products.

The important role played by the pharmaceutical sector in PV in HIC indicates that they are a major stakeholder, which, if adequately engaged, could potentially change the outlook of PV in LMIC. In LMIC, huge volumes of medicines are procured via special donor-supported procedures^79,80^ and safety surveillance is mainly handled via PHP, making the MAH role secondary.^26^ This model is unlikely to change soon, given the important role played by the PHP that enables availability, accessibility and affordability to medicines and vaccines in LMIC; however, it is evident that a stronger engagement of the pharmaceutical sector will enable and support PV development in LMIC. The mechanisms for engaging MAH include the following: mandating safety surveillance and implementing mechanisms to ensure compliance by MAH^60^; providing clear guidance and coherent information on requirements to industry; allocating MAH fees for PV or creating PV-specific fees; harmonisation of PV requirements; and implementing mechanisms for information exchange and knowledge sharing on developing and innovating PV.

### Strategic plan for a systematic approach

This research revealed that the coordination of national PV activities was overall good, with TFP aligning with the national PV plan. Although stakeholder coordination was considered acceptable, it is not optimal. For instance, for many countries participating in this research and in line with current knowledge,^24^ training for HCP remains the main focus of capacity building; training workshops are often the easiest interventions to implement, assess and report. Though essential to address underreporting ADR, this intervention should ideally be needs-driven and conducted within the framework of strengthening the national PV system. A majority of countries involved in this research reportedly had established a PV development plan or roadmap, often guided by the WHO-GBT.^23^ Countries are encouraged to develop and maintain a strategic improvement plan, which is an important tool for PV development. Because countries are at different stages of development, the strengthening of national PV systems requires an ‘end-to-end’ approach, adapted and customised, taking into account the specific national contexts and the country’s ability to develop, absorb and implement an effective PV system. PV systems must also ideally be established within the health and legal regulatory framework of a country to ensure that such systems have the local political and international regulatory support to function properly and with authority. Such a system-driven strategy to PV system strengthening will support the identification of fit-for-purpose solutions to core system challenges, and will reduce silos and duplicity of strengthening interventions.

In view of resource constraints and given current electronic possibilities, a regional and supra-national approach to PV development would be an efficient way forward by pooling human resources and data, harmonising legislations and guidelines, using integrated PV IT platforms, promoting multilateral partnerships and regional collaborations, and data sharing. Examples of these regional approaches are the AU-3S programme^77^ and the SEARN Vigilance Working Group.^78^ Furthermore, in a model similar to the EU regulatory model, in Africa, plans are ongoing to establish the African Medicine Agency as a Specialised Agency of the African Union dedicated to improving access to quality, safe and efficacious medical products in Africa.^81^

### Establish and implement standardised PV processes

The ninth essential element, which cuts accross all PV functions, relates to PV processes and their outcomes. PV processes in LMIC is briefly presented here and extensively addressed in a separate manuscript.^82^ All participating countries had implemented procedures for ADR reporting, investigation and causality assessment and at least one or more critical PV activities, such as signal detection or periodic safety reporting. This was an interesting finding because a majority of participating countries were benchmarked at ML1 (basic) and ML2 (essential), meaning that if these procedures exist, they may not function well. Stakeholders are aware of this gap, because implementing standardised PV processes was the fourth priority area for PV development after training of HCP, signal management and analysis of PV data. Our study revealed that digitalisation of PV activities was one of the factors that has contributed to the recent PV growth in LMIC, resulting notably in an increase in ADR reports, as also reported globally,^83,84^ and thus enabling signal detection and data analysis. Findings from this research support the need to implement simple and efficient procedures to drive PV processes in LMIC. Standardised procedures enable a structured approach to PV activities, with mechanisms to monitor their effectiveness and make continuous improvements. These procedures are essential to achieve the outcomes of PV. There are numerous guidelines, procedures and templates from HIC that can be adapted to LMIC settings and innovative digital solutions for PV, such as VigiBase^®^, VigiLyze^®85^ and ODK,^86^ as well as the capacity-building opportunities proposed by regional collaborations and multilateral partnerships mentioned above. The opportunities are limitless; what is required is ownership and determination.

### Limitations of the research

The sampling of the qualitative study was purposive, therefore potentially subject to bias, because only informants with a good understanding of PV systems and procedures, according to the main author’s judgement, were invited to participate. Secondly, the majority of participants were from LMIC, specifically from the African Region, with only three HIC contributing to the research. Lastly, there was a difference in the number of years of PV experience between qualitative and quantitative, which may explain some divergences in opinions seen in the study.

## Conclusion

Findings from this research show that despite significant progress in PV in LMIC in the last decade, a majority of PV systems in LMIC are still basic or moderately functional, and additional efforts are still required to bring these systems to global PV standards. The nine elements identified in this research must not be developed in a stringent step-by-step sequence. Nonetheless, identifying alternative and continued financing is indispensable for building and sustaining a functional PV system. Stable funding enables the availability of qualified personnel and managerial staff, who, with appropriate legal and political backing, will be able to set up functional and sustainable systems with the necessary structures, governance and processes to achieve the outcomes of PV. Defining a strategy for the national PV system and engaging MAH are decisive steps that should be considered early in the implementation process of the national PV system. Moreover, the element ‘understanding triggers and motivation for PV’ involves a continuous effort to inculcate and foster a PV culture in the country, resulting in a change in mindset.

## Supplemental Material

sj-docx-1-taw-10.1177_20420986251342941 – Supplemental material for Building functional and sustainable pharmacovigilance systems: an analysis of pharmacovigilance development across high-, middle- and low-income countries Supplemental material, sj-docx-1-taw-10.1177_20420986251342941 for Building functional and sustainable pharmacovigilance systems: an analysis of pharmacovigilance development across high-, middle- and low-income countries by Olga Menang, Peter van Eeuwijk, Karen Maigetter, Andrea de Soyres-Kuemmerle, Edinam Agbenu and Christian Burri in Therapeutic Advances in Drug Safety

sj-docx-2-taw-10.1177_20420986251342941 – Supplemental material for Building functional and sustainable pharmacovigilance systems: an analysis of pharmacovigilance development across high-, middle- and low-income countries Supplemental material, sj-docx-2-taw-10.1177_20420986251342941 for Building functional and sustainable pharmacovigilance systems: an analysis of pharmacovigilance development across high-, middle- and low-income countries by Olga Menang, Peter van Eeuwijk, Karen Maigetter, Andrea de Soyres-Kuemmerle, Edinam Agbenu and Christian Burri in Therapeutic Advances in Drug Safety

sj-docx-3-taw-10.1177_20420986251342941 – Supplemental material for Building functional and sustainable pharmacovigilance systems: an analysis of pharmacovigilance development across high-, middle- and low-income countries Supplemental material, sj-docx-3-taw-10.1177_20420986251342941 for Building functional and sustainable pharmacovigilance systems: an analysis of pharmacovigilance development across high-, middle- and low-income countries by Olga Menang, Peter van Eeuwijk, Karen Maigetter, Andrea de Soyres-Kuemmerle, Edinam Agbenu and Christian Burri in Therapeutic Advances in Drug Safety

sj-docx-5-taw-10.1177_20420986251342941 – Supplemental material for Building functional and sustainable pharmacovigilance systems: an analysis of pharmacovigilance development across high-, middle- and low-income countries Supplemental material, sj-docx-5-taw-10.1177_20420986251342941 for Building functional and sustainable pharmacovigilance systems: an analysis of pharmacovigilance development across high-, middle- and low-income countries by Olga Menang, Peter van Eeuwijk, Karen Maigetter, Andrea de Soyres-Kuemmerle, Edinam Agbenu and Christian Burri in Therapeutic Advances in Drug Safety

sj-xlsx-4-taw-10.1177_20420986251342941 – Supplemental material for Building functional and sustainable pharmacovigilance systems: an analysis of pharmacovigilance development across high-, middle- and low-income countries Supplemental material, sj-xlsx-4-taw-10.1177_20420986251342941 for Building functional and sustainable pharmacovigilance systems: an analysis of pharmacovigilance development across high-, middle- and low-income countries by Olga Menang, Peter van Eeuwijk, Karen Maigetter, Andrea de Soyres-Kuemmerle, Edinam Agbenu and Christian Burri in Therapeutic Advances in Drug Safety

## Declarations

**Ethics approval and consent to participate:** This study was approved by the research commission of the Swiss Tropical and Public Health Institute (Swiss TPH, Allschwil, Switzerland). The Ethics Committee of Northwestern and Central Switzerland (EKNZ) waived the need for formal ethics approval (Req-2020-00232) because the research does not fall under the Swiss Human Subject Research Act. The EKNZ reviewed the submitted documents and confirmed that the research project fulfilled the general ethical and scientific standards for research with humans according to Art 51 of the Swiss Human Subject Research Act. For the qualitative study (semi-structured interviews), candidates received an invitation to participate, providing detailed information on the nature, purpose and potential implications of the study, as well as information on confidentiality and the interview procedures. Furthermore, the research did not involve human subjects and did not pose any risks to participants. Therefore, verbal consent to participate in the study and to record the interview was deemed sufficient at the start of each interview. For the quantitative study, survey participants agreed that by filling out the questionnaire, they were consenting to participate in the study.

**Consent for publication:** At the start of the interview, qualitative research participants verbally consented to the use of their information provided in the research for related publications. By filling out the online questionnaire, survey participants consented to the anonymised use of their information provided in the study for related publications.

**Availability of data and materials:** The datasets generated and analysed during the current study are available in the Dryad repository: https://doi.org/10.5061/dryad.2547d7wzs.
