# Supplementary material for: Building functional and sustainable pharmacovigilance systems: an analysis of pharmacovigilance development across high-, middle- and low-income countries
Source: Ther Adv Drug Saf. 2025 Jun 10;16:20420986251342941. doi: 10.1177/20420986251342941 (PMC12185949; doi:10.1177/20420986251342941)
Supplement: sj-docx-1-taw-10.1177_20420986251342941 – Supplemental material for Building functional and sustainable pharmacovigilance systems: an analysis of pharmacovigilance development across high-, middle- and low-income countries [file sj-docx-1-taw-10.1177_20420986251342941.docx]

**Supplemental File 1**

| Interview Guide for qualitative research |
| --- |
| Building functional and sustainable pharmacovigilance systems – an analysis of pharmacovigilance implementation in high-, middle- and low-income countries |
| Welcome and introduction |
| - Introduction of interviewer - Provide the background for the interview and an overview of the PhD research - Express gratitude to the participant for agreeing to contribute to the research - Provide details on the length of the interview - Obtain verbal consent to record interview and include information provided in the research |
| Part 1: Respondent’s role in the national pharmacovigilance system |
| 1. What organization do you work for? 2. What position do you hold within your organization? 3. What are your roles and responsibilities with regards to the national pharmacovigilance (PV) system? 4. Would you say you have any influence on the decisions taken on the functionality of the national PV system? What influence and why so? |
| Part 2: Organization of the national pharmacovigilance system |
| 1. What event(s) triggered the creation of the PV system in your country? 2. When was the PV center or PV system established? 3. In your opinion, how developed is the national PV system in your country? WHO Maturity Level, if available or any other system assessment using standardized tools. 4. Is there a national PV plan that describes the national PV system and the roles and responsibilities of the key stakeholders? 5. What were the strategies that guide the establishment and development of the PV system (e.g. strategic or operational plan)? 6. How is the PV system financed? What is the main source of funding? 7. What were the first challenges you encountered for PV system development? What are the current main challenges encountered? |
| Part 3: Pharmacovigilance and health system development |
| 1. In your opinion, in what way does the healthcare system influence the development of PV? Why so? 2. How is the national PV system integrated into the healthcare system? 3. What is the proportion of the government health budget that is dedicated to PV? 4. How is the healthcare system leveraged to build the national PV system (Service delivery; Health workforce; Financing; Information; Leadership and governance)? 5. In your opinion, would integration of PV at the different levels of the health system (primary, secondary and tertiary) contribute to building more resilient PV systems? If yes: how so? |
| Part 4: Pharmacovigilance and pharmaceutical development |
| 1. In what way does the pharmaceutical development in your country contribute to PV development? Why so? 2. What guidelines are in place to ensure that industry maintains oversight of the safety of authorized medications? 3. How does the pharmaceutical sector interact and collaborate with the national PV programme on PV activities? 4. How would you further leverage the pharmaceutical sector and industry to develop PV in your country? |
| Part 5: Ensuring functional and sustainable pharmacovigilance systems |
| 1. Who are the main PV stakeholders and how is the coordination of PV activities at the national level ensured (create synergy and avoid duplicity)? 2. In your opinion, are the donor and technical agencies’ proposals to strengthen PV often aligned with the national PV plans and the country’s priorities? To what extent? 3. Is the current financing PV activities sufficient? How can you internally generate resources to adequately finance PV activities? 4. What is the main focus of major PV strengthening activities within the country? 5. How much efforts are invested in PV strengthening activities that target training of healthcare workers and national PV experts? 6. What proportion of PV strengthening activities targets more advanced PV activities such as signal detection and management, aggregate data analysis, active safety surveillance? 7. New vaccine introductions are an opportunity to strengthen national PV systems. How have these contributed to the development and enhancement of PV in your country? 8. What other innovations have contributed to the enhancement of PV in your country (e.g. digital tool such as ODK collect)? 9. In your opinion, are the activities currently undertaken to strengthen the national PV system adequate? Why so? 10. Moving forward, what activities would you prioritize to strengthen the national PV system? |
| Closing |
| - Is there anything else you would like to add? - Are there any important issues or topics that have been missed? - Do you have any questions about the interview, the research and the researcher? |
| Thank you for your participation and contribution to this research! |
