# Supplementary material for: Building functional and sustainable pharmacovigilance systems: an analysis of pharmacovigilance development across high-, middle- and low-income countries
Source: Ther Adv Drug Saf. 2025 Jun 10;16:20420986251342941. doi: 10.1177/20420986251342941 (PMC12185949; doi:10.1177/20420986251342941)
Supplement: sj-docx-2-taw-10.1177_20420986251342941 – Supplemental material for Building functional and sustainable pharmacovigilance systems: an analysis of pharmacovigilance development across high-, middle- and low-income countries [file sj-docx-2-taw-10.1177_20420986251342941.docx]

**Supplemental File 2**

| Questionnaire for online survey (Open Data Kit Collect) |
| --- |
| Building functional and sustainable pharmacovigilance systems – an analysis of pharmacovigilance implementation in high-, middle- and low-income countries |
| Introduction |
| Thank you for participating in this survey on Strategies and interventions to build and strengthen pharmacovigilance systems in high, middle and low-income countries. This survey is undertaken in the frame of the doctorate programme of Ms Olga Menang at the Swiss Tropical and Public Health Institute (Swiss TPH) entitled *“Determinants of health systems capacity for effective pharmacovigilance: an analysis of pharmacovigilance implementation in high, middle and low-income countries”.* The objective of the research is to describe key factors of a health system that are essential for implementing an efficient and performing pharmacovigilance (PV) system. The objectives of the proposed questionnaire are to:   - Understand how PV systems in high, middle and low income countries were established and how they are sustained - Describe the influence of health care and pharmaceutical systems on the development of PV - Analyse the capacity for safety data analysis over the last decades in LMIC - To determine if the strategy for establishing and building PV systems in LMIC is appropriate - Propose recommendations on strategies to build and strengthen PV systems in LMIC   Your contribution to this research will provide valuable information on optimal strategies to build more functional and resilient PV systems in LMIC. Your participation in this online survey is completely voluntary and anonymous. Your responses will be confidential and we do not collect identifying information such as your name, email address or IP address. By filling this questionnaire you are consenting to participate in the study and consenting to use the information provided in the research. Filling this this online survey will take approximately 30 minutes. |
| Part A: Respondent’s role in the national pharmacovigilance system |
| A1. What organization do you work for?*  *(*If you do not work for National regulatory authority, PV centre, National immunisation programmeme, sections B (Organisation of the national pharmacovigilance system) does not apply)*  National Regulatory Authority (NRA)  PV centre  National Immunisation Programmeme (NIP)  Donor agencies  Technical Agency (TA)  Industry  Other ___________________  A2. What position do you hold within your organization? _____________________________  A3. How many years of PV experience do you have?  Less than 1 year  2 to 5 years  6 to 10 years  11 to 15 years  More than 16 years  A4. To what extent do you have any influence on the decisions taken on the functionality of the national PV system?  ☐To a Great Extent ☐Somewhat ☐Very Little ☐Not at All |
| Part B: National pharmacovigilance system capacity |
| B1. Has your country’s PV system been assessed using the WHO Global Benchmarking Tool (WHO GBT)?  ☐Yes  ☐No  ☐I don’t know  B2. If yes, what is the maturity level? If No, skip to question B3.  ☐Maturity level 1 ☐Maturity level 2  ☐Maturity level 3 ☐Maturity level 4  B3. Has your country’s PV system been assessed using any other tool (e.g. the Indicator-Based Pharmacovigilance Assessment Tool (IPAT))?  ☐Yes  ☐No  ☐I don’t know  B4. If yes, what was the ranking? If No, skip to question B5.  ☐Minimal (No capacity or have minimal organizational structures and capacity for PV)  ☐Basic (Basic structure in place with policy and legal framework for PV)  ☐Moderate (Capacity to collect and evaluate safety data on the basis of legal and organizational structure)  ☐Advanced (Performing PV systems to detect, evaluate, and prevent medicine safety issues)  B5. Are there sufficiently competent staff (i.e., education, training, skills and experience) assigned to perform vigilance activities?  ☐Yes  ☐No  ☐I don’t know |
| Part C: Pharmacovigilance and health system development |
| C1. The healthcare system influences the development of PV.  ☐Strongly Disagree ☐Disagree  ☐Neither agree nor disagree ☐Agree  ☐Strongly Agree  C2. How many levels are there in healthcare system?  ☐1 ☐2  ☐3 ☐ Other ________________  C3. Is PV integrated at each level?  ☐Yes  ☐No  ☐I don’t know  C4. In your opinion do you think integrating PV at the different levels of the health system has contributed to build a more functional PV system?  ☐Strongly Disagree ☐Disagree  ☐Neither agree nor disagree ☐Agree  ☐Strongly Agree |
| Part D: Pharmacovigilance and pharmaceutical development |
| D1. Pharmaceutical development influences the growth of PV.  ☐Strongly Disagree ☐Disagree  ☐Neither agree nor disagree ☐Agree  ☐Strongly Agree  D2. There are legal provisions and regulations that require manufacturers and/or marketing authorization holders (MAH) to set up a vigilance system of their medical products and periodically report vigilance data to the NRA.  ☐Not implemented (there are no legal provisions and regulations)  ☐Ongoing implementation (the NRA is taking steps to establish legal provisions and regulations)  ☐Partially implemented (legal provisions and regulations are in early implementation phase so no consolidated results exist yet).  ☐Implemented (legal provisions and regulations are actually implemented and enforced).  D3. To what extent is the pharmaceutical sector involved with the national PV programme or activities aimed at developing the national PV system?  ☐To a Great Extent ☐Somewhat  ☐Very Little ☐Not at All  D4. What proportion of the fees levied on the pharmaceutical sector is dedicated to PV activities?  ☐Very High ☐High  ☐ Considerable ☐Very low  ☐None  D5. Very Low Do you think directing some of the fees to support PV activities will help to fund PV and build more sustainable PV systems?  ☐Strongly Disagree ☐Disagree  ☐Neither agree nor disagree ☐Agree  ☐Strongly Agree |
| Part E: Strategies to build and strengthening national pharmacovigilance systems |
| F1. Is there a strategic plan or operational plan that describes the process to strengthen and continually improve the PV system?  ☐Yes  ☐No  ☐I don’t know  F2. If there are no strategic or implementable national PV strengthening plans, briefly describe how strengthening is accomplished at the national level.________________________________  F3. In your opinion how effective is the coordination of stakeholder at the national level ensure synergy and avoid duplicity of efforts.  ☐Very Good ☐Good  ☐Acceptable ☐Poor  ☐Very Poor  F4. How is the PV system financed?  ☐Primarily donor ☐Donor and country  ☐Primarily national dedicate budget ☐Others _____________________  F5. Without external funding are you able to conduct activities aimed at building the PV system?  ☐Yes  ☐No  ☐I don’t know  F6. To what extent are donor and technical agencies’ proposals to strengthen PV aligned with the national PV plans and the country’s priorities?  ☐To a Great Extent ☐ Somewhat  ☐Very Little ☐Not at All  F7. Tick the top 3 priority areas for strengthening the national PV system  ☐Training of healthcare workers on adverse event reporting  ☐Training of healthcare workers adverse event investigation  ☐Training of national drug safety review committee on causality assessment  ☐Signal detection, evaluation and management  ☐Developing standard operating procedures for pharmacovigilance activities  ☐Aggregate analysis of PV data e.g. periodic safety update reports  ☐Active safety surveillance  ☐Other __________________________________________  F8. What proportion of PV strengthening activities targets more advanced PV activities such as signal detection and management, aggregate data analysis, active safety surveillance?  ☐None ☐Less than 10%  ☐Less than 25% ☐More than 50%  ☐More than 80%? ☐I don’t know  F9. New vaccine introduction represents opportunities to strengthen the national PV system. Tick ways in which vaccine introductions have contributed to the development of PV (more than one answer is possible)  ☐ Increase adverse event reporting rate  ☐Development of PV tools such as reporting forms  ☐Electronic reporting and management of adverse events  ☐Capacity for advanced PV activities such as active surveillance  ☐Analysis of PV data  ☐Other______________________________  F10. The development of software tools for collecting and processing information on adverse events (e.g. ODK™) have contributed to improve reporting and analysis of PV data. Tick ways in which these tools have contributed to the development of PV (more than one answer is possible)  ☐ Increase adverse event reporting rate  ☐ Adequate handling and management of adverse events  ☐Signal detection and management  ☐Analysis of aggregate PV data  ☐Other________________________________________  F11. Is the current approach to strengthen the PV system adequate?  ☐Strongly Disagree ☐Disagree  ☐Neither agree nor disagree ☐Agree  ☐Strongly Agree  F12. List at least one recommendation you would make to improve the current PV strengthening strategy ___________________________________________________________________ |
| Thank you for your participation! |
