# Supplementary material for: Building functional and sustainable pharmacovigilance systems: an analysis of pharmacovigilance development across high-, middle- and low-income countries
Source: Ther Adv Drug Saf. 2025 Jun 10;16:20420986251342941. doi: 10.1177/20420986251342941 (PMC12185949; doi:10.1177/20420986251342941)
Supplement: sj-docx-3-taw-10.1177_20420986251342941 – Supplemental material for Building functional and sustainable pharmacovigilance systems: an analysis of pharmacovigilance development across high-, middle- and low-income countries [file sj-docx-3-taw-10.1177_20420986251342941.docx]

**Supplemental File 3**

| Codebook for qualitative research | | | |
| --- | --- | --- | --- |
| Building functional and sustainable pharmacovigilance systems – an analysis of pharmacovigilance implementation in high-, middle- and low-income countries | | | |
| Category 1 : Respondent’s role in the national pharmacovigilance (PV) system | | | |
| Codes | Code description | Sub codes | Assumption/comment |
| Organisation | Where the respondent works | Technical and financial partner (TFP) | Major PV stakeholder |
|  |  | National regulatory authorities (NRA) |  |
|  |  | National Immunization Programs (NIP) |  |
| Position in organisation | Number of years of PV experience | PV expert | Senior positions can influence PV system functionality |
|  |  | No PV expert |  |
| Roles and responsibilities | Has roles and responsibilities vis a vis the PV system | PV responsibilities | None |
| Influence on PV decisions | Can influence decisions taken on the PV system | PV decision | Can drive PV decisions |
|  |  | PV implementation |  |
| Category 2: Organization of the national pharmacovigilance system | | | |
| Codes | Code description | Sub codes | Assumption/comment |
| Triggers for establishing PV | What major event triggered the creation of the PV system | Vaccine introduction | PV created due to evolution in global requirements and new products |
|  |  | Drug introduction |  |
|  |  | World Health Organisation (WHO) program |  |
| PV centre establishment | PV system younger than 5 years | PV <10 years | The older the system, the more mature or advanced |
|  | PV system older than 5 years | PV >10 years |  |
| PV maturity | WHO maturity level | Maturity I | The more mature the more performance |
|  |  | Maturity II |  |
|  |  | Maturity II |  |
|  |  | Basic PV |  |
|  |  | Moderate PV |  |
|  |  | Advanced PV |  |
| Description of PV system including roles and responsibilities | Organisation chart with roles and responsibilities of major stakeholders | PV framework | Clear description of PV system with stakeholder’s R and R clearly explained ensures coordination and collaboration |
|  |  | Legal basis |  |
|  |  | PV guidelines |  |
| PV strategic plan | Strategic plan describing development of PV system | PV strategy | A strategic plan with guiding principles on steps to develop PV may result in a more structured approach and therefore more efficient development |
|  |  | No PV strategy |  |
|  |  | PV operational plan |  |
|  |  | Other plans |  |
| PV system financing | Mainly donor driven | External financing | Countries with dedicated internal funds can build more sustainable systems |
|  | Mainly internally dedicated PV budget | Internal financing |  |
|  | Hybrid type of funding | Mixed financing |  |
| PV challenges | Challenges when system was established | Initial challenges | Are the challenges same as before or evolved? A developing system should see challenges evolve as well |
|  | Challenges today | Current challenges |  |
| Category 3: Pharmacovigilance and health system development | | | |
| Codes | Code description | Sub codes | Assumption/comment |
| Influence of health system on PV - reasons | To understand how PV is influenced by the health system |  | PV in most low- and middle-income countries (LMIC) is built on health systems; the more developed the health system, the more developed is the PV system |
| Integration of PV in health system | How PV is integrated at different levels of the health system | PV at all levels | Integration at different levels of the health system would grow PV |
|  |  | Focal points at district level |  |
|  |  | Focal points in health facilities |  |
|  |  | Parallel systems |  |
| Proportion of health budget dedicated to PV | If there is a dedicated budget for PV | PV budget | PV would be more functional with dedicated budget |
| Further leveraging health system for PV | How can the different health system building blocks be further utilised to grow PV | Leverage health system for PV |  |
| Integrating PV at different health system levels to develop PV | Collaboration between PV and public health programmes (PHP) | Collaboration with PHP | Effectively integrating PV at different health system levels can help grow PV without huge additional resources. |
|  | PV coordinated at national level | PV at central level |  |
|  | PV integrated at different levels of the system | PV at intermediate level |  |
|  | PV integrated at different levels of the system | PV at lower levels |  |
| Category 4: Pharmacovigilance and pharmaceutical development | | | |
| Codes | Code description | Sub codes | Assumption/comment |
| Influence of pharma growth on PV development | Influence of pharma growth on PV development  Influence of pharma on PV | Industry contribution | The more developed the pharma sector, the more developed PV |
|  |  | No industry contribution |  |
|  |  | Local Pharma contribution to PV |  |
| Guidelines for pharma | Any guidance or regulations in place for pharma | Guidelines for pharma | Clear guidance enables performance |
|  |  | Mandatory reporting |  |
|  |  | Manufacturer responsibility |  |
| Interaction with pharma | Ways in which pharma interact with national PV system | PV activities |  |
|  |  | Data quality |  |
|  |  | PV training |  |
|  |  | Weak pharma collaboration |  |
|  |  | Conflict interest |  |
|  |  | Strong pharma collaboration |  |
| Further leveraging pharma sector for PV | How can the pharm sector be further used to develop PV e.g. fees? | Improve risk management plans (RMP) | Pharma sector can be a strong source of revenue to fund and sustain PV |
|  |  | Improve case quality |  |
|  |  | Alerts |  |
|  |  | Suspension |  |
| Category 5: Building functional and sustainable PV systems | | | |
| Codes | Code description | Sub codes | Assumption/comment |
| Stakeholder coordination | Main national and global PV actors and national coordination of PV activities | PV stakeholders | Mapping stakeholders and coordinating PV activities result in more efficient PV systems |
|  |  | Stakeholder coordination |  |
| Donors alignment to national PV development plan | Donors aligned to PV strengthening plan if one exists | Donors aligned | PV systems are mainly donor driven and donors can influence activities to development of PV, not necessarily aligned to PV plan |
|  |  | Donors influence |  |
| PV financing | Is the current PV funding sufficient and how can sustainable financing be ensured | Sufficient funding | Assigning some MAH fees to PV activities can ensure sustainable financing for PV |
|  |  | Insufficient funding |  |
|  |  | Plans to leverage fees for PV |  |
| Focus of PV strengthening | Focus of PV strengthening activities | Strengthening priority | The more advanced the system, the more advanced the activities |
|  |  | Training on reporting |  |
|  |  | Signal detection |  |
|  |  | Safety/risk communication |  |
|  |  | Periodic safety update reports (PSUR) |  |
|  |  | RMP |  |
|  |  | Active surveillance |  |
|  |  | Electronic reporting |  |
| Efforts invested in training HCP on reporting on AEs | Focus of strengthening is sensitization and training of healthcare professionals (HCP) on reporting | Basic activities | As systems growth they must evolve to more complex systems with more advanced PV activities |
|  |  | Significant effort |  |
| Efforts invested in more advanced PV activities | Investments in more advanced activities | Advanced activities |  |
|  |  | Little or no advanced activities |  |
| Influence of vaccine introduction on PV development | Impact of vaccine introductions on PV growth | Vaccines improve PV | Vaccine introductions are opportunities to develop PV. Huge strengthening with each vaccine introduction |
|  |  | Resource allocation |  |
|  |  | PV awareness |  |
| Impact of other innovations e.g. ODK collect on PV | Impact of ODK on PV growth | ODK improves PV | Increased in reporting thanks to ODK |
| Adequacy of PV development strategy | Is the PV strategy adequate? | PV strategy | If the strategy to build PV is adapted, reasonable and feasible. |
|  |  | PV operational plan |  |
|  |  | PV strategy adequate |  |
| Priorities for developing PV | What activities are prioritized for PV strengthening | Signal detection | Strategic planning with strategies define according to needs, capacity and development of PV |
|  |  | Risk communication |  |
|  |  | Active surveillance |  |
|  |  | Electronic reporting |  |
|  |  | Stakeholder collaboration |  |
|  |  | Continuous training |  |
|  |  | Improved collaboration |  |
