# Supplementary material for: Building functional and sustainable pharmacovigilance systems: an analysis of pharmacovigilance development across high-, middle- and low-income countries
Source: Ther Adv Drug Saf. 2025 Jun 10;16:20420986251342941. doi: 10.1177/20420986251342941 (PMC12185949; doi:10.1177/20420986251342941)
Supplement: sj-docx-5-taw-10.1177_20420986251342941 – Supplemental material for Building functional and sustainable pharmacovigilance systems: an analysis of pharmacovigilance development across high-, middle- and low-income countries [file sj-docx-5-taw-10.1177_20420986251342941.docx]

**Supplemental File 5**

**Checklist for Mixed Methods Research (MMR) Manuscript Preparation and Review^1^**

**Building functional and sustainable pharmacovigilance systems – an analysis of pharmacovigilance development across high-, middle- and low-income countries**

| Topic | Guide Questions/Description | Reported on page number |
| --- | --- | --- |
| Rational and description of MMR design | Provide a clear statement of the study purpose | 6 |
|  | Explicitly describe the MMR design in accordance with Creswell’s (2015) typology and use a diagram to illustrate the relationship and sequence of qualitative and quantitative research components | 6 |
|  | Justify why the MMR design is appropriate for meeting the study purpose | 6 |
| Transparency in describing method details | Describe the study population(s) and sample(s; e.g., who, what, how many) | 6 – 7 |
|  | Describe the sampling procedures (including inclusion and exclusion criteria, recruitment) | 6 – 7 |
|  | Describe qualitative data collection processes (how often data were collected, who collected the data, what kind of data collection instruments were used, how data were recorded—e.g., notes, transcripts) | 7 |
|  | Describe qualitative data analysis processes (coding, single or multiple coders, replication logic, credibility) | 7 – 8 |
|  | Describe quantitative data analysis procedures (missing data and how they are handled, statistical tests used) | 8 |
| Integration of qualitative and quantitative research components | Interpret qualitative analysis results with appropriate quotes if necessary | 12 – 16 |
|  | Interpret quantitative analysis results in consideration of statistical significance, selection bias, and threats to validity | 12 – 16 |
|  | Compare qualitative and quantitative results | 12 – 16 |
|  | Address divergences and inconsistencies between qualitative and quantitative results | 19, 21 |

*A*dapted from: Lee S-YD, Iott B, Banaszak-Holl J, et al. Application of Mixed Methods in Health Services Management Research: A Systematic Review. *Med Care Res Rev* 2021; 79: 331-344. DOI: 10.1177/10775587211030393.
